# Triplex DNA-based Bioanalytical Platform for Highly Sensitive Homogeneous Electrochemical Detection of Melamine

**DOI:** 10.1038/s41598-017-04812-4

**Published:** 2017-07-03

**Authors:** Xiaojuan Liu, Mengmeng Song, Feng Li

**Affiliations:** College of Chemistry and Pharmaceutical Sciences, Qingdao Agricultural University, Qingdao, 266109 People’s Republic of China

## Abstract

Melamine detection has attracted much attention since the discovery of the damage of melamine to human health. Herein, we have developed a sensitive homogeneous electroanalytical platform for melamine detection, which is relied on the formation of triplex molecular beacon integrated with exonuclease III (Exo III)-mediated signal amplification. The formation of triplex molecular beacon was triggered by the recognition and incorporation of melamine to the abasic (AP) site contained in the triplex stem. The stem of the triplex molecular beacon was designed to have a protruding double-strand DNA, which can be recognized and hydrolyzed by Exo III for releasing methylene blue (MB)-labeled mononucleotide. These released MB molecules exhibit high diffusivity toward indium tin oxide electrode with negative charge, thus producing a significantly increased electrochemical response. Taking advantages of the high binding affinity of the DNA triplex structure containing AP sites towards melamine and the unique features of Exo III, this sensing platform is capable for sensitive and selective melamine assay with a detection limit as low as 8.7 nM. Furthermore, this strategy shows good applicability for melamine assay in real samples. Therefore, this strategy broadens the application of triplex DNA and presents a new method for sensitive detection of melamine.

## Introduction

Melamine, which is a triazine heterocyclic organic compound, has been widely used in the production of plastics, coatings, flame retardants, melamine resins, and other products^1^. In the past years, melamine has been illegally and unethically added to food and animal feeds to increase its apparent crude protein content, because customary Kjeldahl protein analysis approach fails to distinguish the nitrogen sources of protein from that of non-protein^2^. However, the intake of melamine for human and animals could lead to the formation of insoluble complexes in kidney and subsequent tissue injury^3^. Therefore, much effort has been focused on developing reliable methods for detecting melamine in food, whereas little attention has been paid on monitoring melamine contained in the food chain, such as food crops grown by nitrogenous fertilizers, animal meat fed by melamine-mixed feeds. As those melamine-contaminated foods are easily ignored, it is of great importance to establish effective and reliable assay for monitoring melamine at the source of the food chain.

Traditional melamine detection methods include mass spectroscopy (MS)^4^, gas chromatography/mass spectrum (GC/MS)^5^, high performance liquid chromatography (HPLC)^6^ and so on. Although these approaches can achieve high sensitivity and selectivity, they are usually dependent on expensive instruments, time-consuming sample pretreatment, careful calibration, and skilled manpower, which greatly limit their utility in routine melamine determination. To overcome these problems, various alternative methods have been explored for detecting melamine, such as colorimetric assay^7–10^, fluorometric assay^11, 12^, surface enhanced Raman scattering (SERS)^13^, immunoassay approaches^14^, electrochemical methods^15, 16^. Among them, electrochemical method, as a useful analytical technology, has been extensively employed because of its outstanding merits of inexpensive, fast detection, convenient operation procedures, high sensitivity, and compatibility with micro-manufacturing techniques^17, 18^. In particularly, recently developed homogeneous electrochemical methods open a promising way for the design of immobilization-free electrochemical biosensors^19–21^. In these biosensors, the recognition of analyte and subsequent reaction are performed in homogeneous solution instead of the interface between the solution phase and electrode surface. This homogeneous reaction can increase the reliability and reproducibility of the electrochemical biosensor owing to the decreased steric hindrance effect on electrode surface. Furthermore, compared with traditional heterogeneous electrochemical strategies, the homogeneous electroanalytical methods avoid the time-consuming and tedious steps for the modification of electrode, which makes the experimental procedures more convenient and much simpler. Using these homogeneous electrochemical strategies, various targets, such as DNA, biological molecules, and metal ions, etc. have been successfully detected^19–30^. For example, Hsing and co-workers reported several homogeneous electroanalytical approaches for detecting Hg^2+^ and DNA^22–24^. Our group developed various homogeneous electroanalytical approaches for sensitive analysis of ATP, activities of DNA methyltransferase, alkaline phosphatase, and human telomerase^25–29^.

Recently, triplex DNA has attracted much attention because of its indispensable in many biological processes, such as gene translation, gene expression, DNA transcription, and replication^31^. The triplex structure is constructed through binding the third DNA strand to the double-helical DNA via Hoogstern hydrogen bonds^32–34^. The formation of triplex structure can be regulated by external triggers such as target molecules, pH, and metal ions^35–37^, making it a potentially powerful tool for numerous applications in biological chemistry and medicine^38, 39^. For example, triplex DNA has been utilized as recognition motifs in the design of various biosensing platforms and DNA nanomachines through the target-induced triplex DNA formation and dissociation^40, 41^. Apart from the sequence-specific recognition of double-stranded DNA, the incorporation of the third DNA strand into the stem of molecular beacons to form hairpin-like DNA structure is an alternative strategy for the construction of biosensors^42–44^. For instance, triplex molecular beacons have been utilized to design melamine aptamer by introducing an abasic (AP) site into the triplex stem^43, 44^. In this aptamer, the integrated AP site could provide a hydrophobic vacancy for binding melamine through hydrogen bonding interaction between thymine and melamine. Based on this aptamer, this group has developed selective fluorescence biosensors for melamine based on Mg^2+^-dependent DNAzyme and silver nanoclusters, respectively^43, 44^. Most recently, Wang and Lin *et al*. have constructed a sensitive and selective electrochemical biosensor for melamine determination by designing AP site into triplex DNA, in which four DNA strands can form a T-shaped DNA nanostructure in the presence of melamine. This biosensor exhibits excellent performance for melamine detection and can be used for testing the migration of melamine from melamine bowl^45^.

In this work, we present a homogeneous electroanalytical strategy for sensitive assay of melamine based on the formation of triplex molecular beacon and exonuclease III (Exo III)-assisted signal molecules release. This approach employs a hairpin DNA (HP) and a 3′-methylene blue (MB) labeled DNA (MB-DNA) containing AP sites as the melamine recognition part, Exo III as a tool enzyme, and an indium tin oxide (ITO) electrode with negative charge as the working electrode. In the presence of melamine, the formation of triplex molecular beacon occurs and then MB molecules are released from MB-DNA by Exo III, resulting in a significantly increased electrochemical signal of MB. Taking advantages of the high selectivity of the DNA triplex structure containing AP sites towards melamine and the unique features of Exo III, this proposed strategy is capable of detecting melamine with high sensitivity and selectively.

## Results and Discussion

### Design of Melamine Detection Strategy

**Figure 1:**
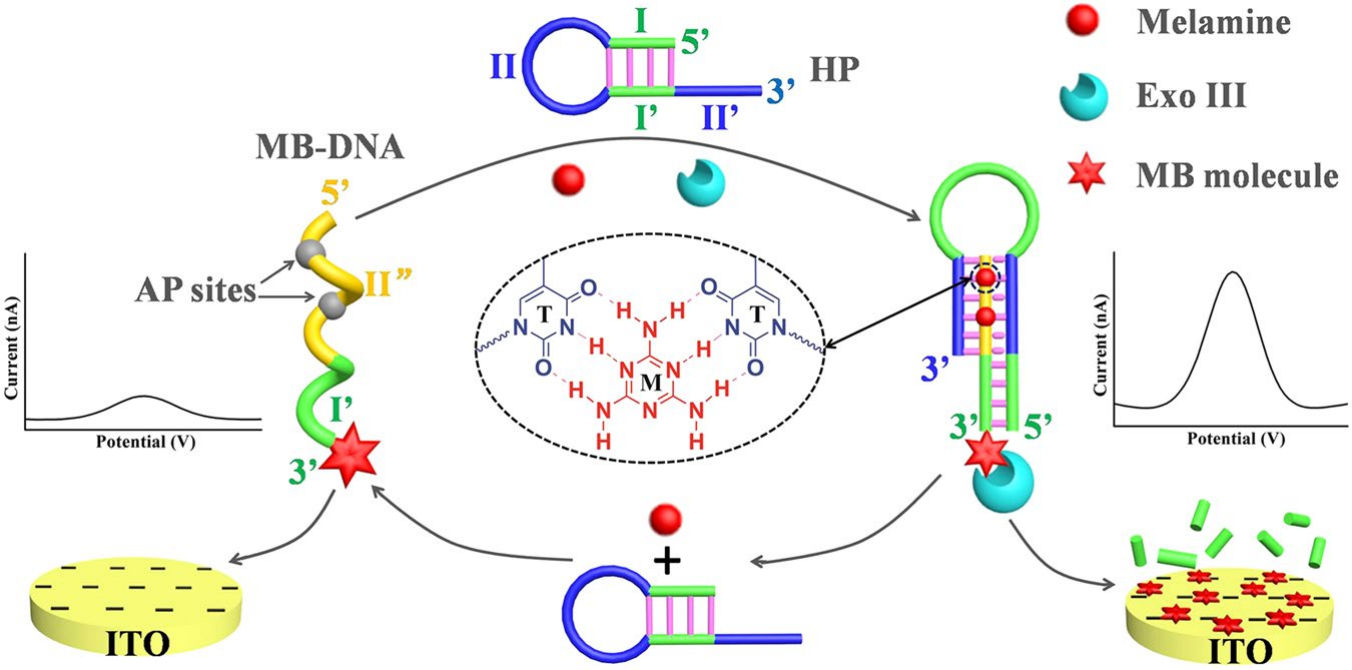
Principle of the homogeneous electroanalytical strategy for melamine assay.

Figure 1 schematically illustrates the strategy for sensitive homogeneous electroanalysis of melamine, which is based on a simple system of HP, MB-DNA, and Exo III. HP is an ingeniously designed hairpin DNA, which consists of four sequential domains, namely I, II, I′, and II′. The base sequence of domain I is complementary to that of domain I′, while domains II and II′ are short poly(dT) sequences. So, HP alone has a hairpin structure with a protruded fragment (domain II′). MB-DNA is a 3′-methylene blue (MB) labeled DNA, which contains two sequential domains: a poly(dA) sequence with two AP sites (domain II″) and a same base sequence to domain I′. Exo III, which displays sequence dependence, can catalyze the stepwise removal of 3′ mononucleotides from blunt or recessed 3′-termini of double-stranded DNA^46–49^. In the absence of melamine, MB-DNA cannot reconfigure HP because of the limited stability of triplex stem with AP sites. Thus, MB-DNA maintains single strand structure and gives a very weak electrochemical signal of MB, because both negatively charged MB-DNA and ITO electrode have strong electrostatic repulsion. However, in the presence of melamine, melamine can locate in the AP sites of the triplex DNA structure and form a stable T-melamine-T structure through hydrogen bonding interaction^43, 44^. Thus, MB-DNA can hybridize with HP to form a molecular beacon with a triplex stem (domains II, II′, and II″) and a protruding double strand (domains I and I′). This duplex with blunt 3′ termini is then recognized and cleaved by Exo III, leading to the digestion of MB-DNA in the protruding duplex. This digestion process releases MB-labeled mononucleotide, melamine, and HP. The released melamine and HP can hybridize with another MB-DNA and trigger a new digestion process. The released MB-labeled mononucleotide, which has less negative charge than that of MB-DNA, possesses higher diffusivity toward ITO electrode owing to the reduced electrostatic repulsion. As a result, a remarkably increased electrochemical signal would be obtained. Therefore, this strategy realized the sensitive detection of melamine in a “signal-on” mode.

### Feasibility Study

**Figure 2:**
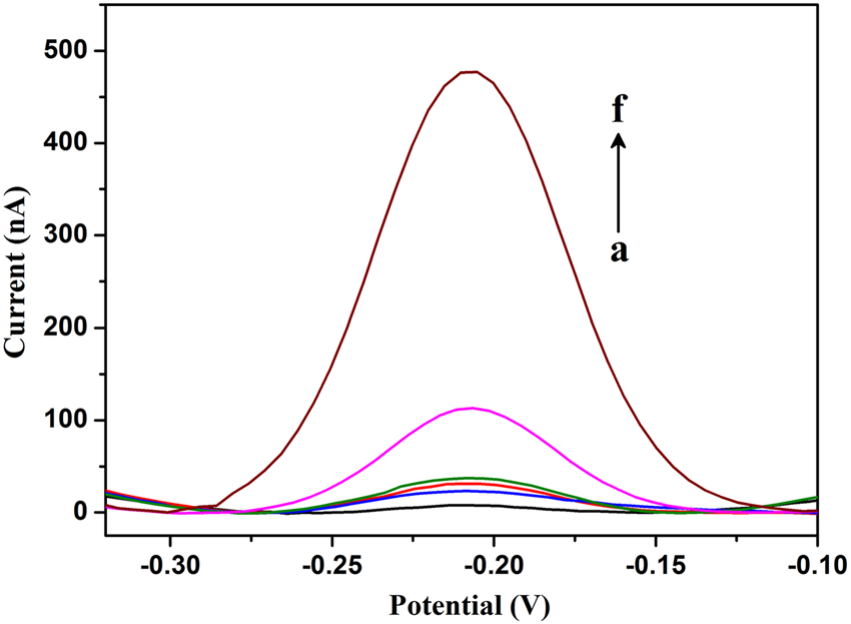
Differential pulse voltammograms (DPV) of the sensing system in the presence of: (**a**) melamine; (**b**) HP, MB-DNA, and melamine; (**c**) MB-DNA only; (**d**) MB-DNA and Exo III; (**e**) HP, MB-DNA, and Exo III; (**f**) HP, MB-DNA, melamine, and Exo III.

To verify the feasibility of melamine assay, a series of control experiments were preformed and their electrochemical responses were recorded. As shown in Fig. 2, no electrochemical signal was detected from the solution of melamine (curve a) due to the absence of electrochemical signal molecule. In the presence of HP, MB-DNA, and melamine, a negligible DPV current of MB molecule was observed (curve b), and the intensity of the current was almost the same as that obtained from MB-DNA solution (curve c), indicating that the negatively charged MB-DNA and the triplex DNA structure were hard to reach the surface of ITO electrode owing to the strong electrostatic repulsion between them. Additionally, when only Exo III was mixed with MB-DNA, the DPV current was almost unchanged (curve d), because the single strand MB-DNA can resist the digestion of Exo III. Nevertheless, because of the existence of equilibrium between different molecular conformations of HP, small portion of HP may be in a single-stranded conformation, which would hybridize with MB-DNA and produce blunt 3′ termini, causing the cleavage of some MB-DNA by Exo III. As a result, addition of HP and Exo III to MB-DNA solution led to a slightly increased DPV current (curve e). However, a remarkably increased DPV current of MB was obtained (curve f) when melamine was added into the reaction system, indicating the successful hybridization between HP and MB-DNA, as well as the subsequent digestion of MB-DNA by Exo III.

### Optimization of Assay Conditions

**Figure 3:**
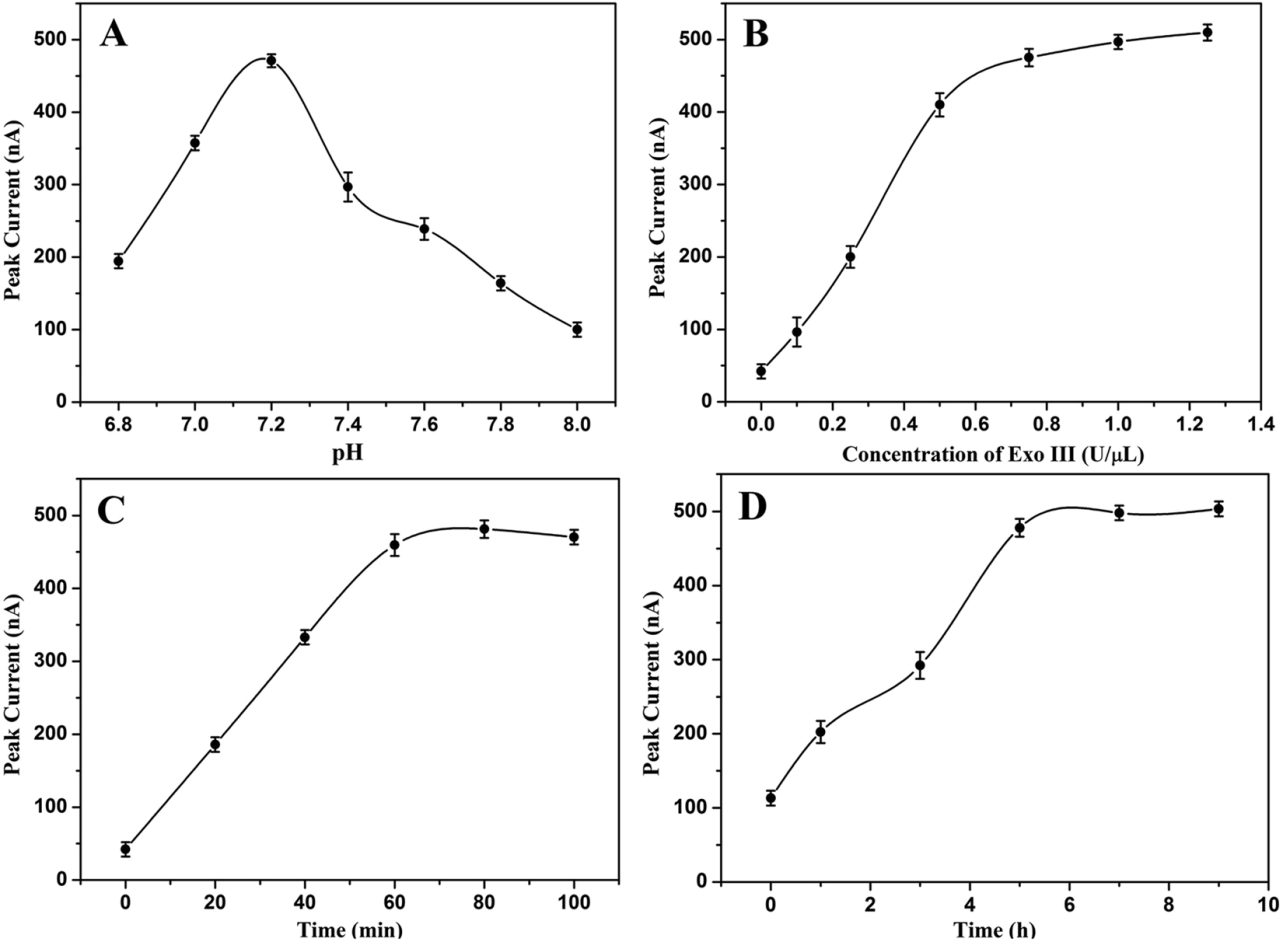
DPV peak current observed under (**A**) different pH values: 6.8, 7.0, 7.2, 7.4, 7.6, 7.8, and 8.0; (**B**) different Exo III concentrations: 0, 0.1, 0.25, 0.5, 0.75, 1, and 1.25 U/μL; (**C**) different reaction time of Exo III: 0, 20, 40, 60, 80, and 100 min; (**D**) different reaction time of melamine: 0, 1, 3, 5, 7, and 9 h. The concentration of melamine was 50 μM. The error bars represent the standard deviation of three measurements.

In this melamine sensing strategy, the electrochemical signal was generated by the released MB-labeled mononucleotide, which originated from MB-DNA. To ensure the high sensitivity, the amounts of MB-DNA should be sufficient, thus 1 μM MB-DNA was added into the reaction solution during the following experiments. Furthermore, as the formation of Hoogsteen TA**·**T triplex structure is sensitive to pH value, the influence of pH was optimized by analyzing the electrochemical signals in the presence of 50 μM melamine. As shown in Fig. 3A, the intensity of DPV peak current obviously increased when the pH value increased from 6.8 to 7.2, but further increasing the pH value resulted in dramatically decreased peak intensities. The highest DPV peak current was acquired at pH = 7.2, which was chosen as the optimal pH value. To achieve the best analytical performance, the concentration of Exo III, the reaction time of Exo III cleavage, and melamine incorporation were also optimized, respectively. Figure 3B reveals that a higher DPV signal was obtained when a higher concentration of Exo III was adopted. But the DPV peak current increased slowly when the concentration was higher than 0.75 U/μL. Thus, 0.75 U/μL was selected as the optimal Exo III concentration. Figure 3C shows the effect of reaction time of Exo III cleavage on this melamine sensing strategy. Obviously, the DPV peak currents increased remarkably as the time of Exo III cleavage extended to 60 min. Afterward, the DPV peak currents did not change much with increased cleavage time, suggesting that the release of MB-labeled mononucleotide from the MB-DNA was almost finished at enzymatic reaction time of 60 min. Similarly, obviously increased DPV peak currents were observed with the extension of the melamine incorporation time and almost reach the saturation value at 5 h (Fig. 3D). Therefore, 60 min and 5 h were adopted as the optimal Exo III cleavage time and melamine incorporation time, respectively.

### Sensitivity of Melamine Assay

**Figure 4:**
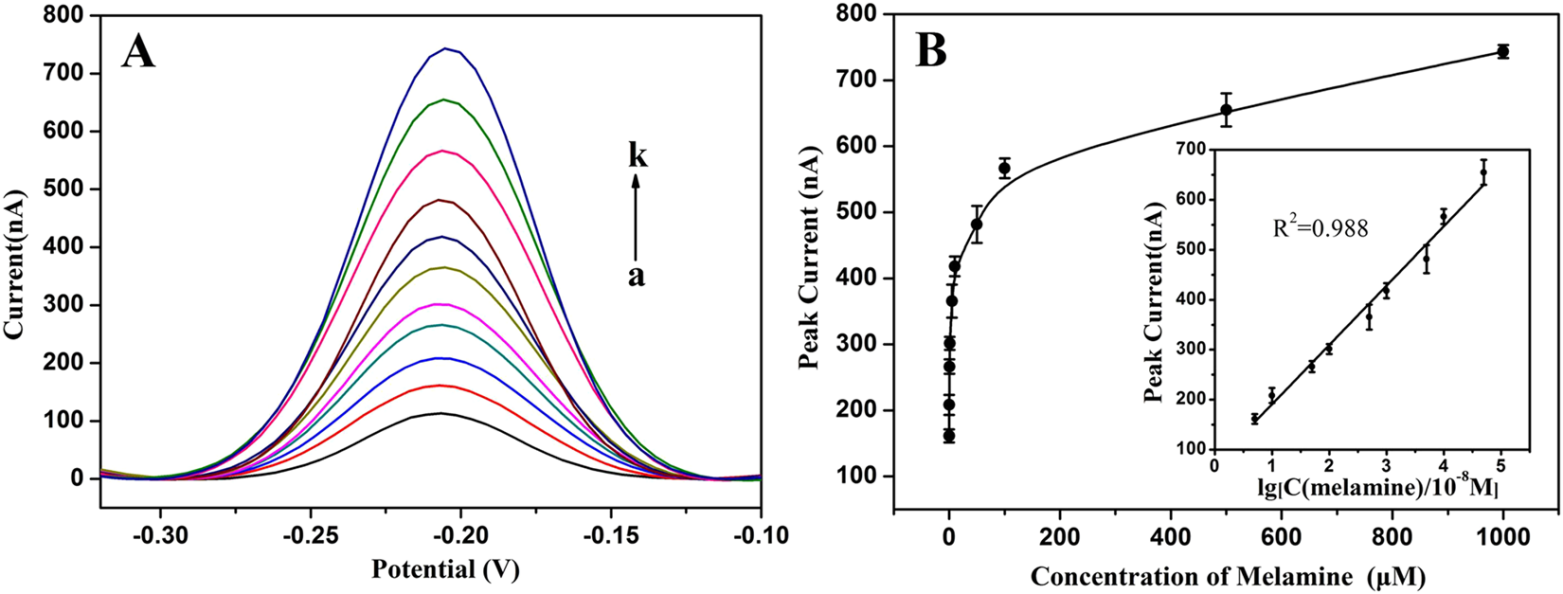
(**A**) DPV currents of the sensing system after adding melamine with different concentrations: (a–k) 0, 0.05, 0.1, 0.5, 1.0, 5.0, 10, 50, 100, 500, and 1,000 μM, respectively. (**B**) Calibration curve corresponding to the peak current versus melamine concentration ranging from 50 nM to 1,000 μM. Inset: the linear relationship between DPV peak current and melamine concentration ranging from 50 nM to 500 μM. The error bars represent the standard deviation of three measurements.

The performance of this approach for melamine assay was investigated by analyzing the electrochemical response of the sensing system towards different concentrations of melamine. As shown in Fig. 4A, the DPV current of MB molecule was sensitive to melamine concentration and gradually increased with the increase of melamine concentration. The relationship between melamine concentration and the intensity of DPV peak current is presented in Fig. 4B. Obviously, melamine concentration ranging from 0 to 1000 μM could be directly measured by this sensing strategy. Furthermore, the inset of Fig. 4B shows a good linear relationship between the intensity of peak current and the logarithm of melamine concentration ranging from 50 nM to 500 μM. The correlation equation was determined to be *i*_*p*_ = 118.7 log C_melamine_ + 72.0 (R^2^ = 0.988), where, *i*_*p*_ is the intensity of peak current (nA), C_melamine_ is the melamine concentration (10^−8^ M). The detection limit towards melamine was estimated to be 8.7 nM (1.1 ppb), which is lower than that of the above mentioned biosensors (Table 1) and the maximum level permitted by China (1 ppm for infant formulas), USA and European Union (2.5 ppm), and India (1 ppm for infant formulas), respectively^50, 51^.

**Table 1:** Comparison of the analytical performance of the present method with that of previously reported methods.

Strategy for detection	Method	Detection limit	Ref.
Colorimetric assay based on uracil-5-carboxylic acid and 2,4,6-trinitrobenzenesulfonic acid tailored AuNPs	Colorimetry	5 ppb	51
Colorimetric assay based on citrate-stabilized AuNPs	Colorimetry	25 ppb	52
Colorimetric assay based on nitroaniline-modified Ag NPs	Colorimetry	100 ppb	53
Colorimetric detection based on cysteamine-modified AuNPs	Colorimetry	600 ppb	54
Fluorescence detection based on aggregation-induced emission-active tetraphenylethene	Fluoremetry	600 ppb	2
Indirect competitive ELISA method based on monoclonal antibodies	ELISA	500 ppb	14
Ultrasound-assisted extractive electrospray ionization mass spectrometry (EESI-MS)	EESI-MS	270 ppb	50
Ag nanorod SERS substrate based detection	SERS	100 ppb	13
electrochemical sensor based on its copper complex	Electrochemistry	250 ppb	55
Triplex DNA-based biosensing platform	Electrochemistry	1.1 ppb (8.7 nM)	This work

### Selectivity of Melamine Analysis

**Figure 5:**
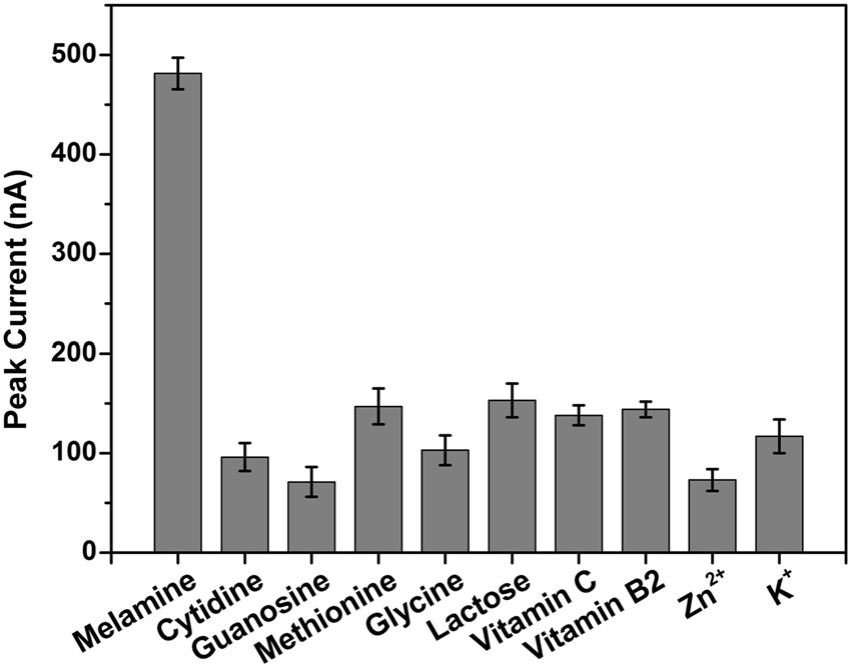
Comparison of DPV peak currents in the presence of melamine, cytidine, guanosine, methionine, glycine, lactose, vitamin C, vitamin B_2_, Zn^2+^, and K^+^. The concentrations of melamine are 50 μM and other substances are 100 μΜ. The error bars represent the standard deviation of three measurements.

The selectivity of the proposed strategy for melamine assay was evaluated by testing the electrochemical response of this sensing system toward several potential interfering substances, such as cytidine, guanosine, methionine, glycine, lactose, vitamin C, vitamin B_2_, Zn^2+^, and K^+^ (Fig. 5). Compared with the significant increase of the DPV peak current upon melamine addition, there were only weak electrochemical signals observed for most other species. Therefore, this homogeneous electroanalytical strategy exhibited excellent selectivity for melamine detection and has great potential for real sample analysis application.

### Real Sample Analysis

To investigate the applicability of this electroanalysis method for real sample assay, melamine in animal feed was tested by using this strategy. As melamine concentration in the animal feed was lower than the detection limit of this method, standard addition experiments were preformed. The electrochemical responses of the spiked samples were analyzed and the results are listed in Table 2. Analytical results showed that the melamine concentrations in the spiked real samples were statistically close to the concentration of melamine added and their recoveries are ranging from 90% to 97%. Although these recoveries are lower than 100%, they are still in the recovery range permitted by Chinese National Standards (60~120%, GB/T27404-2008). Thus, random deviation of measurement is speculated to be the main reason for the inconsistency between melamine concentration detected and added. Moreover, the complex matrix such as animal feed has no evident influence on this homogeneous electroanalytical strategy, suggesting the good anti-interference capability and the potential application of this method for the analysis of melamine in real samples.

**Table 2:** Recovery data for feed samples added with different concentrations of melamine (n = 5).

Sample number	Melamine added (μM)	Melamine detected (μM)	Recovery (%)	RSD (%)
1	5	4.5	90	5.7
2	10	9.7	97	4.5
3	50	46.0	92	7.2

## Conclusions

In summary, a homogeneous electroanalysis platform for sensitive melamine assay was developed based on melamine-triggered formation of triplex molecular beacon and Exo III-assisted signal amplification. Taking advantage of the high binding affinity of AP site-containing triplex structure towards melamine and the unique properties of Exo III, numerous MB-labeled mononucleotides were released, which results in significantly increased electrochemical signals. All results consistently confirmed that the new electrochemical platform was applicable for melamine detection with high sensitivity and selectively. Additionally, this strategy exhibits good potential for the analysis of melamine in real samples. Thus, this strategy broadens the application of triplex DNA and presents a new strategy for the sensitive and selective detection of melamine.

## Methods

### Reagents

Hydrochloric acid (HCl), tris(hydroxymethyl) aminomethane (Tris), MgCl_2_, NaCl, ZnCl_2_, ascorbic acid were obtained from Sinopharm Chemical Reagent Co., Ltd. (Shanghai, China). Exo III, guanosine monophosphate, cytidine monophosphate, glycine, and lactose were obtained from Shanghai Sangon Biotechnology Co., Ltd. (Shanghai, China). Melamine was purchased from Aladdin Reagents Co., Ltd. (Shanghai, China). All these chemicals were used without further treatment. All solutions were prepared by using ultrapure water (resistivity  18.2 MΩ cm) obtained from a Milli-Q water purification system (Millipore Corp., Bedford, MA, USA). The HPLC-purified DNA probes were obtained from Shanghai Sangon Biotechnology Co., Ltd. (Shanghai, China) and their sequences were listed as follows:

HP: 5′-GTC TGT TTT TTT TCT CAG ACT CTT TTT TTT-3′

MB-DNA: 5′-AGA AA X AA X ACA GAC-MB-3′

X denotes the AP site of spacer C3.

The MB-DNA and HP were directly used and diluted in 50 mM Tris-HCl (pH = 7.2, containing 100 mM NaCl, 5 mM MgCl_2_) to give the stock solution of 10 µM.

### Melamine Assay

The melamine assay was performed in 50 µL Tris-HCl reaction buffer containing 1 μM HP, 1 µM MB-DNA, and target melamine with different concentrations. This reaction solution was incubated at 25 °C for 5 h, followed by the addition of 37.5 U Exo III and incubation at 37 °C for 60 min before the electrochemical measurements.

### Sensing Melamine in Pig Feed

Pig feed, a mixed powder mainly consists of corn, soybean, bran, fish meal, minerals (such as ZnSO_4_, KI, FeSO_4_, CuSO_4_), and vitamins (such as vitamin A/B_2_/C/D/E/K), was chosen as the real sample matrix to evaluate the practical application of the as-proposed approach for melamine detection. Firstly, 1.0 g pig feed was mixed with 20 mL Tris-HCl reaction buffer in a centrifuge tube, and the mixture was ultrasonicated and shaken for 10 min, respectively. After repeating this process for two times, the mixture was centrifugated at 15,000 rpm for 10 min, then the supernatant was collected and further filtered with 0.45 μm filter before melamine assay. Finally, different concentrations of melamine were added into the feed samples to prepare the melamine spiked samples. Each concentration was repeated for 5 times, and the result was an average of 5 measurements.

### Electrochemical Measurement

Differential pulse voltammetric (DPV) measurements were carried out on an Autolab Electrochemical Workstation (Metrohm, Switzerland) with a conventional three-electrode system: an ITO electrode as the working electrode, a platinum wire as the counter electrode, and an Ag/AgCl electrode as the reference electrode. It should be noted that ITO electrode with negative charge was obtained by using a previously reported pretreatment method^29^.
